# Supplementary material for: Anti-Inflammatory and Pro-Differentiating Properties of the Aryl Hydrocarbon Receptor Ligands NPD-0614-13 and NPD-0614-24: Potential Therapeutic Benefits in Psoriasis
Source: Int J Mol Sci. 2021 Jul 13;22(14):7501. doi: 10.3390/ijms22147501 (PMC8304622; doi:10.3390/ijms22147501)
Supplement: Supplementary file 1 [file ijms-22-07501-s001.zip › ijms-1282003-supplementary.pdf]

Supplementary Materials:

Gene		Oligonucleotide sequences (5′-3′)	Amplicon size	Accession number
**AhR**	Forward	ATCACCTACGCCAGTCGCAAG	137 bp	NM_001621.4
	Reverse	AGGCTAGCCAAACGGTCCAAC		
**BD-2 (beta-defensin2)**	Forward	GATGCCTCTTCCAGGTGTTTTT	75 bp	NM_001205266.1
	Reverse	GGATGACATATGGCTCCACTCTT		
**CASP14**	Forward	ACATCGCCTACCGACATGATC	110 bp	NM_012114.3
	Reverse	CCGGGTCACCTCTGTCAGAA		
**CYP1A1**	Forward	CTGGAGACCTTCCGACACTCTT	84 bp	NM_000499.5
	Reverse	GTAAAAGCCTTTCAAACTTGTGTCTCT		
**CYP1B1**	Forward	AACGTACCGGCCACTATCAC	139 bp	NM_000104.3
	Reverse	CCACGACCTGATCCAATTCT		
**FLG (Filaggrin)**	Forward	GAAGACAAGGATCGCACCAG	76 bp	NM_002016.2
	Reverse	ATGGTGTCCTGACCCTCTTG		
**GAPDH**	Forward	TGCACCACCAACTGCTTAGC	198 bp	NM_001289746
	Reverse	GGCATGGACTGTGGTCATGAG		
**IL-1α**	Forward	CGCCAATGACTCAGAGGAAGA	120 bp	NM_000575
	Reverse	AGGGCGTCATTCAGGATGAA		
**IL-1β**	Forward	CTGAGCTCGCCAGTGAAATG	77 bp	NM_000576.2
	Reverse	TTTAGGGCCATCAGCTTCAAA		
**IL-6**	Forward	AGCCACTCACCTCTTCAGAACG	141 bp	NM_000600
	Reverse	GGTTCAGGTTGTTTTCTGCCAG		
**IL-8**	Forward	CTTGGCAGCCTTCCTGATTTC	168 bp	NM_000584
	Reverse	TTCTGTGTTGGCGCAGTGTG		
**INV (Involucrin)**	Forward	ACCCATCAGGAGCAAATGAAA	67 bp	NM_005547.4
	Reverse	GCTCGACAGGCACCTTCTGGC		
**NOQ1**	Forward	GGATTGGACCGAGCTGGAA	140 bp	NM_000903.2
	Reverse	AATTGCAGTGAAGATGAAGGCAAC		
**NRF-2**	Forward	CTTGGCCTCAGTGATTCTGAAGTG	124 bp	NM_001313903
	Reverse	CCTGAGATGGTGACAAGGGTTGTA		
**S100A7**	Forward	CTTCTACTCGTGACGCTTCC	205 bp	NM_002963.4
	Reverse	AATTTGTGCCCTTTTTGTCA		

Supplemental Table S1. Primers used for the Real time RT-PCR analysis

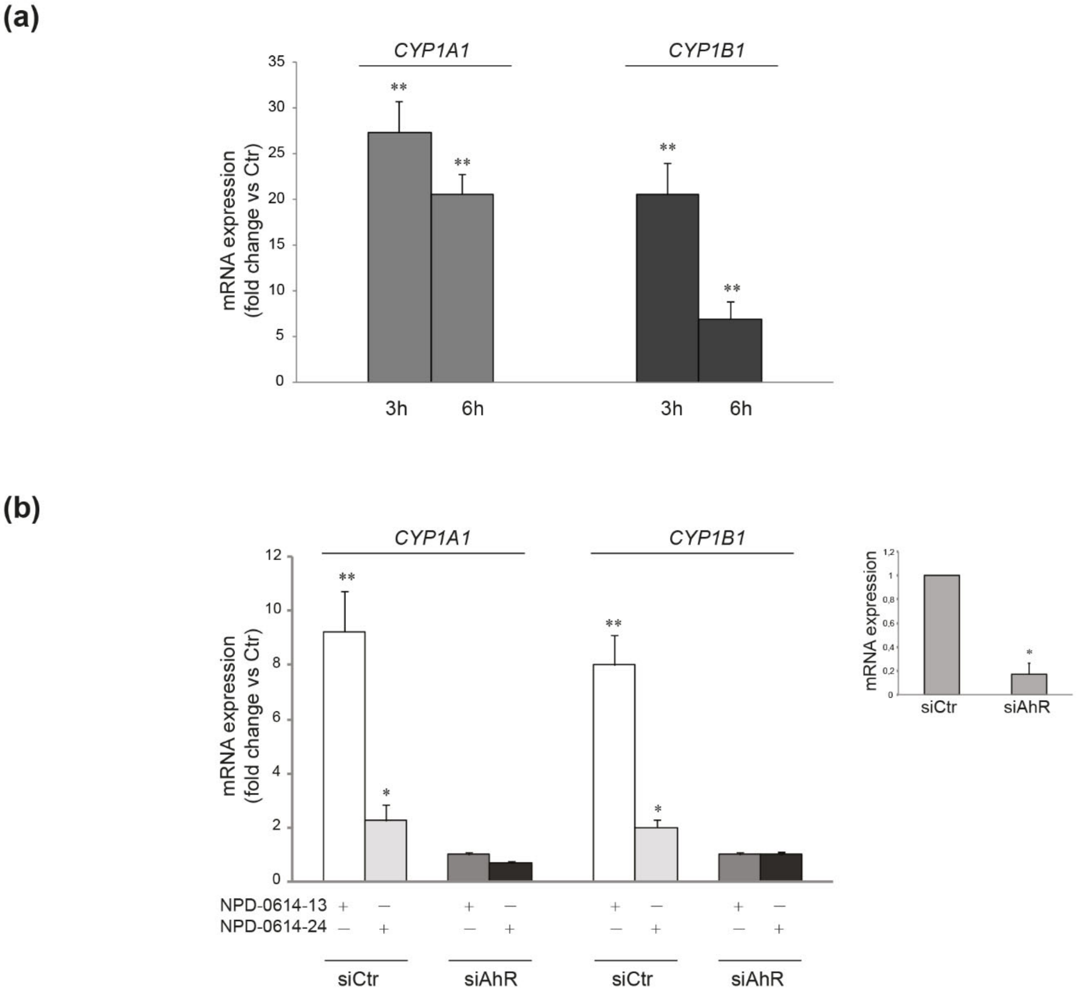

Figure S1

Figure S1. (a) Activation of AhR signaling in response to FICZ in NHKs. Quantitative real time PCR analysis of CYP1A1 and CYP1B1 in NHKs treated with FICZ (100 nM) for 3-6 h. All mRNA values were normalized against the expression of GAPDH and were expressed relative to untreated control cells. Results are expressed as the fold change respect to untreated control cells (*p<0.05, **p<0.01 vs untreated control). **(b)** Activation of AhR signaling in response to NPD-0614-13 and NPD-0614-24 in NHKs. Quantitative real time PCR analysis of CYP1A1 and CYP1B1 in NHKs transfected with siRNA specific for AhR (siAhR) or siCtr and stimulated with NPD-0614-13 and NPD-0614-24 (25 μM) for 24 h. Data represent the mean ± SD of three independent experiments. Results are expressed as the fold change respect to untreated control cells (*p < 0.05, **p < 0.01 vs untreated control).

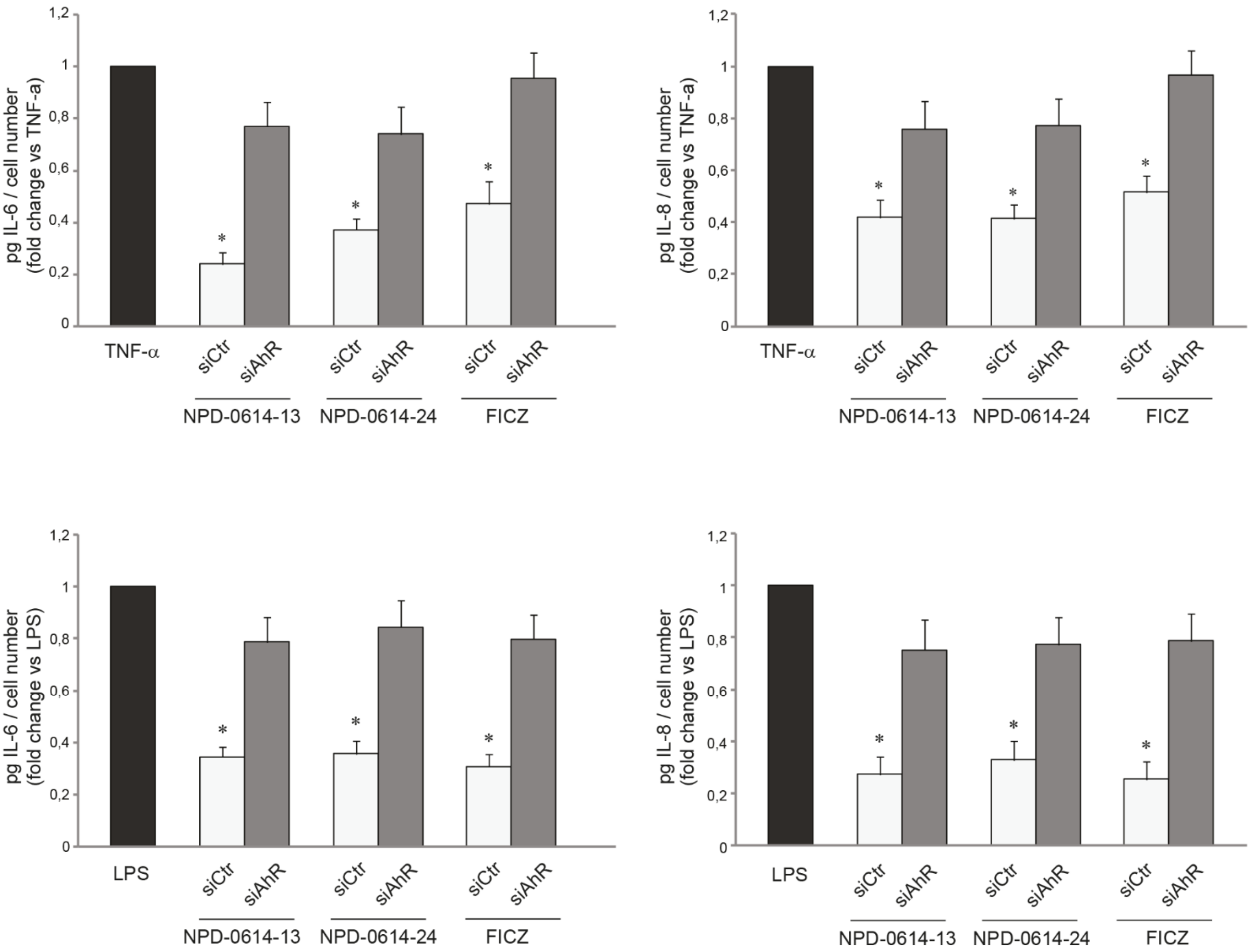

Figure S2

Figure S2. The anti-inflammatory effect of NPD-0614-13 and NPD-0614-24 required AhR activation. IL-6 and IL-8 quantitation by ELISA in NHKs transfected with AhR siRNA or siCtr and treated with TNF-α (20 ng/ml) or LPS (10 μg/ml) in the presence or absence of NPD-0614-13, NPD-0614-24 (25 μM) and FICZ (100 nM) for 24 h. Data represent the mean ± SD of three independent experiments. Results are expressed as the fold change respect to untreated control cells (*$p < 0.05$, **$p < 0.01$ vs untreated control).
